# Supplementary material for: Using a multi-strain infectious disease model with physical information neural networks to study the time dependence of SARS-CoV-2 variants of concern
Source: PLoS Comput Biol. 2025 Feb 14;21(2):e1012778. doi: 10.1371/journal.pcbi.1012778 (PMC11828408; doi:10.1371/journal.pcbi.1012778)
Supplement: Attachment — Submitted filename: review_comments.docx [file pcbi.1012778.s014.docx]

**Review comments**

Li et al., *Using a multi-strain infectious disease model with physical information neural networks to study the time dependence of SARS-CoV-2 variants of concern*

Thank you very much for the opportunity to review this manuscript.

In this study, the authors describe and fit a Physics-Informed Neural Network (PINN) model to epidemiological data of two SARS-CoV-2 variants in British Columbia, Canada. The proposed algorithm allows them to fit the time-varying rate parameters of a traditional SIR-type model to data and provides a longitudinal look at the effects of different virological, environmental, and sociological factors on the spread of SARS-CoV-2 variants.

In general, I find this to be an interesting application of neural networks to epidemiological data. However, I have some concerns regarding both specific details and the overall structure of the manuscript.

Before I list my comments on specific sections of the manuscript, I will briefly list my main criticisms, which I feel should definitely be resolved before publication:

- The model structure is not explained in deep enough detail to follow and evaluate the results shown.
- Multiple concepts are left unexplained to the reader or mentioned once and then never again. Examples include the data from Italy mentioned in the introduction, the contraction factor and temporal interpolation points in the discussion.
- The discussion is currently very short and does not link up well with the rest of the manuscript. In particular, it doesn’t discuss the specific insights gained for the spread of SARS-CoV-2 variants in British Columbia, and neither is this new algorithm contrasted with more traditional analyses based on “pure” compartmental models.
- Data and code used for the analysis are not shared and the reasons for this are not discussed. Reference 35, which ostensibly links to SARS-CoV-2 data in British Columbia, currently points to <https://www.example.com>.
- The whole manuscript should be edited for clarity of language and grammatical consistency. Some sentences are currently somewhat nonsensical (lines 213-214 as an example). These should be either clarified or removed.

Here is a list of comments on specific sections (some repetition possible):

**Specific comments:**

- **Author summary:** This section reads a bit strangely. Some of the wording is excessively flowery, e.g., “profound fusion of deep neural networks with epidemiological models,” but that’s my personal taste. However, the sentence “Our research findings reveal a fascinating aspect of disease transmission dynamics the diversity of control measures in different regions” should be re-written for grammatical correctness.
- **Introduction, lines 78-80:** It’s not clear what role the data from Italy plays in the rest of the manuscript, as it isn’t mentioned anymore. This should be explained either in the methods or results section or removed entirely.
- **Introduction, lines 86-93:** I would remove the mention of Sections 2, 3, and 4. This is more confusing than helpful since no section headings are actually named that way.
- **Methods, lines 96-121:** This section should be expanded. The equations in (1) are somewhat confusing since the sum dS/dt is divided by N, as is the first term of dI_i_/dt, but neither dI_i_^C^/dt nor the terms containing γ_i_(t) are divided by N. This could be resolved by defining explicitly all the relevant rates and compartments in the model, either in the text, or as a table. This should also include the units of each rate and compartment.
- **Methods, lines 113-116:** I would mention here also that R_e_ decreases as S(t) decreases.
- **Methods, lines 134-139:** Is there a specific reason to have 2n independent neural networks that each fit a single parameter as opposed to doing the same thing in a single neural network with 2n outputs?
- **Methods, lines 185-186:** Please explain the types of layers used in these neural networks.
- **Methods, lines 213-214:** “When we compared the estimated parameters with the data, we discovered that the transmission rates are more in line with the fluctuations in the data.” This sentence needs to be clarified or removed. As it stands, I’m not sure what it’s trying to say. More in line than what?
- **Methods, lines 217-218:** “Additionally, the removal rates are more similar, indicating that the recovery time of two VOIs is more consistent.” Like the above comment, more similar/consistent than what?
- **Results, lines 229-238:** These sentences could be moved to the discussion.
- **Results, lines 267-270:** Though the model predictions fit the real data quite well, it’s difficult to judge exactly *how well* they fit since Fig. 9 and Fig. 10 are dominated by big peaks early on. Consider a version of Fig. 9 and Fig. 10 that is zoomed in further on the testing data. Also, though the predictions are very stable to fluctuations in parameter values, they seem to systematically overestimate the testing data. Please comment on the possible reasons for this.
- **Discussion, lines 339-344:** The authors discuss the importance of the contraction factor. Please introduce this factor either in the methods section, or, very briefly, in the discussion.
- **Discussion, line 340-344:** Setting aside the exact nature of the contraction factor, it seems too much to claim a “strong connection” between this factor and population size, when the study only used regionally aggregated data in a single province of Canada and does not systematically compare the results from areas of different population sizes. However, it is a possible limitation of the proposed algorithm and should therefore be moved to the limitations section.
- **Discussion, lines 345-351:** Like the contraction factor, the concept of temporal interpolation points should be introduced before this discussion.
- **Discussion:** Please compare a more traditional epidemiological model, such as an SIR-type model fit using least squares, and the model proposed in this paper, especially on the topics of accuracy, robustness and computational complexity. Furthermore, discuss the concrete results obtained via VOCs-INN in the context of the SARS-CoV-2 epidemic in Canada. To this end, the lines 239-253 could be moved to the discussion. In addition to being important information for epidemiologists and public health professionals, addressing these points would help to link up the discussion with the introduction.
- **All figures:** Since there are multiple visually similar figures, their figure legends should be expanded where appropriate to include more information on the specific model and analysis shown.
- **Fig. 1 and elsewhere:** Is it expected that γ(t) is nearly completely stable over the entire study duration?
- **Fig. 5:** This figure should either be further clarified in the figure legend or simplified. It’s not clear what the different point sizes and colors mean.
- **Fig. 6:** This figure could use an expanded legend.
- **Fig. 7:** Panels a and b are not necessary in my opinion, as all the information is contained in panel c.
- **Fig. 7-12:** Consider adding some important interventions presented in Fig. 5 as vertical lines to better understand some of the fluctuations.
- **Fig. 9:** The legend refers to shaded areas, which are however not visible by eye without zooming in excessively. If I understand correctly, this is due to the shaded areas being extremely small. It would be good to point out that these areas are very small in the legend to avoid confusion.
- **Fig. S2, S4-S9:** Please provide a key for the abbreviations used as figure titles (IH, FH, NH, VCH, VIH). Their meanings can be guessed using Fig. S1, but it should be part of the figure legends.
