# Supplementary material for: Using a multi-strain infectious disease model with physical information neural networks to study the time dependence of SARS-CoV-2 variants of concern
Source: PLoS Comput Biol. 2025 Feb 14;21(2):e1012778. doi: 10.1371/journal.pcbi.1012778 (PMC11828408; doi:10.1371/journal.pcbi.1012778)
Supplement: Attachment — Submitted filename: Response to Reviewer 2.docx [file pcbi.1012778.s015.docx]

**PCOMPBIOL-D-24-01438, Using a multi-strain infectious disease model with physical information neural networks to study the time dependence of SARS-CoV-2 variants of concern,**

**by Li, Chen and Liu et al**

**Authors’ responses to the comments and suggestions of Reviewer #2**

We first of all would like to thank you for careful reading of our manuscript and for all the constructive critiques and helpful suggestions, which have been instrumental improved the quality of our manuscript. In the revised manuscript, we have carefully considered each of your points. Below are our detailed responses and the modifications we have made.

Your comments are in italic and our responses are in normal text, for the ease of reading.

1. **Response to [***The model structure is not explained in deep enough detail to follow and evaluate the results shown.***]**‌

· **Answer [**We fully concur with your suggestion. In response, we have revised the manuscript to include a more thorough explanation of the model structure. Specifically, Table 1 has been added to clarify each compartment and parameter of the model.**]**

2. ‌‌**Response to [***Multiple concepts are left unexplained to the reader or mentioned once and then never again. Examples include the data from Italy mentioned in the introduction, the contraction factor and temporal interpolation points in the discussion.***]**‌

· **Answer [**Thank you for your feedback. We have addressed the concerns regarding concepts. Specifically, we have moved the Italian data to the comparison section between VOCs-INN and least squares algorithm for clearer illustration (Line 356-388). Additionally, we have added further explanations for the contraction factor and temporal interpolation points in the discussion section.**]**

3. ‌**Response to [***The discussion is currently very short and does not link up well with the rest of the manuscript. In particular, it doesn’t discuss the specific insights gained for the spread of SARS-CoV-2 variants in British Columbia, and neither is this new algorithm contrasted with more traditional analyses based on “pure” compartmental models*.**]**‌

· **Answer [**Thank you for your feedback. We have revised the discussion section to address your concerns. Specifically, we have included a comparative analysis using data from Italy, highlighting the advantages of the VOCs-INN algorithm over the traditional least squares method. Additionally, we have incorporated a detailed discussion on the insights gained regarding the spread of SARS-CoV-2 variants in British Columbia.**]**

4. ‌**Response to [***Data and code used for the analysis are not shared and the reasons for this are not discussed. Reference 35, which ostensibly links to SARS-CoV-2 data in British Columbia, currently points to <https://www.example.com>.***]**‌

· **Answer [**We apologize for not sharing the data and code used in the analysis and for the incorrect reference link in Reference 35. To address these issues, We have shared our code in Python on Github for readers to verify, <https://github.com/jluorganization/VOCs-INN>. And we have provided links to the COVID-19 datasets from the BC province and Italy, <https://bccdc.shinyapps.io/genomic_surveillance/>, <https://github.com/CSSEGISandData/COVID-19>.**]**

5. ‌**Response to [***The whole manuscript should be edited for clarity of language and grammatical consistency. Some sentences are currently somewhat nonsensical (lines 213-214 as an example). These should be either clarified or removed.***]**‌

· **Answer [**We apologize for any lack of clarity in the language and grammatical inconsistencies in our manuscript. We have revised the entire document to improve clarity and coherence, with special attention to the sentences on lines 213-214, which have been clarified as needed.**]**

**specific sections**

6. ‌**Response to [*Author summary:*** *This section reads a bit strangely. Some of the wording is excessively flowery, e.g., “profound fusion of deep neural networks with epidemiological models,” but that’s my personal taste. However, the sentence “Our research findings reveal a fascinating aspect of disease transmission dynamics the diversity of control measures in different regions” should be re-written for grammatical correctness*.**]**‌

· **Answer [**Thank you very much for your feedback on the Summary. I appreciate your insights and acknowledge that the wording might have been a bit overly elaborate in some parts. Your preference for a more straightforward tone is noted, and we will take it into consideration for future writing.**]**

7. ‌**Response to [*Introduction, lines 78-80:*** *It’s not clear what role the data from Italy plays in the rest of the manuscript, as it isn’t mentioned anymore. This should be explained either in the methods or results section or removed entirely.***]**‌

· **Answer [**We apologize for the lack of clarity in our initial presentation. The data from Italy serves as a crucial component in our analysis. Specifically, in the discussion section, we utilize the Italian COVID-19 daily incidence data to compare the effectiveness of our new algorithm with traditional approaches. This comparison allows us to demonstrate the superior performance of our proposed method in accurately modeling and predicting disease transmission dynamics. Given its importance in validating our methodology, we believe it is essential to retain this dataset in the manuscript. **]**

8. ‌**Response to [*Introduction, lines 86-93:*** *I would remove the mention of Sections 2, 3, and 4. This is more confusing than helpful since no section headings are actually named that way.***]**‌

**Answer [**Thank you for pointing out this issue. In the revised manuscript, I have removed all references to these specific section numbers and instead used descriptive phrases to guide the reader through the manuscript.**]**

9. ‌**Response to [*Methods, lines 96-121:*** *This section should be expanded. The equations in (1) are somewhat confusing since the sum dS/dt is divided by N, as is the first term of dI_i_/dt, but neither dI_i_^C^/dt nor the terms containing γ_i_(t) are divided by N. This could be resolved by defining explicitly all the relevant rates and compartments in the model, either in the text, or as a table. This should also include the units of each rate and compartmen*t.**]**‌

**Answer [**We apologize for the confusion in the equations and have revised the text to expand on the model description. We have explicitly defined all relevant rates and compartments, including their units, both in the text and in a new table. The equations have been updated to ensure consistency.**]**

10. ‌**Response to [*Methods, lines 113-116:*** *I would mention here also that R_e_ decreases as S(t) decreases.***]**‌

**Answer [**We appreciate your suggestion. We will add the statement that R_e decreases with the decrease of S (t) in an appropriate position in the article to improve the content of the paper.**]**

11. ‌**Response to [*Methods, lines 134-139:*** *Is there a specific reason to have 2n independent neural networks that each fit a single parameter as opposed to doing the same thing in a single neural network with 2n outputs?***]**‌

**Answer [** Thank you very much for your careful review on our paper. Regarding the issue you mentioned about designing 2n independent neural networks, in fact, this design is based on several key considerations: Firstly, the 2n outputs are relatively independent, so we believe that adapting each parameter independently to a neural network can better capture their respective feature relationships. If we use a single neural network with 2n outputs, the input information may not be sufficient to effectively learn the detailed features of each output, which may affect the accuracy of parameter estimation. Secondly, in order to output 2n parameters simultaneously, the width and depth of a single neural network need to be increased, which may lead to more complex structures and cause problems such as gradient vanishing, especially in back-propagation. Splitting into 2n independent neural networks helps alleviate this problem, as each network can focus on one output, simplifying the back-propagation process and improving training stability and efficiency.**]**

12. ‌**Response to [*Methods, lines 185-186:*** *Please explain the types of layers used in these neural networks.***]**‌

**Answer [**Regarding the type of neural network structure you mentioned, we use a simple feed forward neural network, where each layer is a fully connected layer (i.e., each neuron is connected to all neurons in the previous layer, and the output is the result of a linear combination of inputs plus an activation function). The number of layers in the network and the number of neurons in each layer are empirically adjusted based on the total population in the data.**]**

13. ‌**Response to [*Methods, lines 213-214:*** *“When we compared the estimated parameters with the data, we discovered that the transmission rates are more in line with the fluctuations in the data.” This sentence needs to be clarified or removed. As it stands, I’m not sure what it’s trying to say. More in line than what?***]**‌

**Answer [**We have carefully considered your feedback. I have revised the sentence for clarity: 'In the process of comparing the estimated parameters with actual data, we observed a high consistency between the transmission rates and data fluctuations.' This should address the ambiguity you pointed out. **]**

14. ‌**Response to [*Methods, lines 217-218:*** *“Additionally, the removal rates are more similar, indicating that the recovery time of two VOIs is more consistent.” Like the above comment, more similar/consistent than what?***]**‌

**Answer [** We appreciate your suggestion. I have revised the sentence for clarity: 'Additionally, the removal rates are more similar, indicating that the recovery time of two VOIs is more consistent.' **]**

15. ‌**Response to [*Results, lines 229-238:*** *These sentences could be moved to the discussion.***]**‌

**Answer [**I will move these sentences to the discussion section of the revised version and rewrite the discussion.**]**

16. ‌**Response to [*Results, lines 267-270:*** *Though the model predictions fit the real data quite well, it’s difficult to judge exactly how well they fit since Fig. 9 and Fig. 10 are dominated by big peaks early on. Consider a version of Fig. 9 and Fig. 10 that is zoomed in further on the testing data. Also, though the predictions are very stable to fluctuations in parameter values, they seem to systematically overestimate the testing data. Please comment on the possible reasons for this.***]**‌

**Answer [**We totally agree with your suggestion. In response to increase comparative experiments, we have made the following modifications and additions.

Firstly, regarding the degree of fit between model predictions and real data, we do recognize that relying solely on Figures 9 and 10 may be difficult to accurately determine the quality of the fit, as the early data peaks in the figures are relatively large and may mask other details. In order to more accurately demonstrate the performance of the model on the test data, we have added enlarged versions of Figures 9 and 10 in the auxiliary materials, which focus on the test data area so that readers can see more clearly the corresponding relationship between the model predictions and the real data.

In addition, to quantify the degree of fit predicted by the model, we used the coefficient of determination R² as the evaluation metric. The R² value can comprehensively reflect the closeness between the predicted values of the model and the actual observed values, providing strong data support for our conclusion.

We have had a thorough discussion regarding the issue you mentioned that the model predictions seem to systematically overestimate the test data. We used the continuous growth data of cases in Italy to make similar predictions in the discussion section to demonstrate the predictive ability of VOCs-INN. Calculate the coefficient of determination R²=0.9078 for the prediction stage**.]**

17. ‌**Response to [*Discussion, lines 339-344:*** *The authors discuss the importance of the contraction factor. Please introduce this factor either in the methods section, or, very briefly, in the discussion.***]**‌

**Answer [**Thank you for carefully reviewing our manuscript and emphasizing the need for a clearer introduction to the contraction factor. We appreciate your constructive feedback and have added an explanation of the reduction factor in our discussion.**]**

18. ‌**Response to [*Discussion, line 340-344:*** *Setting aside the exact nature of the contraction factor, it seems too much to claim a “strong connection” between this factor and population size, when the study only used regionally aggregated data in a single province of Canada and does not systematically compare the results from areas of different population sizes. However, it is a possible limitation of the proposed algorithm and should therefore be moved to the limitations section.***]**‌

**Answer [**We understand and agree with your viewpoint regarding the relationship between contraction factor and population size. Indeed, based on data from only one province in Canada, claiming a "strong correlation" between shrinkage factors and population size without systematic comparison of different population size regions appears to be insufficiently rigorous, and the wording in the manuscript may be ambiguous.

The setting of contraction factor is a common preprocessing step in training neural networks, especially in regression tasks. By scaling the data, training effectiveness can be improved, numerical stability can be enhanced, and model convergence can be accelerated. In this study, we used a custom contraction factor and empirically adjusted it based on the total population of different regions. For example, the contraction factor set within the province of British Columbia is 10^4, while in the IH region it is 10^2. This adjustment aims to ensure better numerical stability of the model on different regional datasets. However, based on these observations, our initial conclusion about the "strong correlation" between contraction factors and population size does appear imprecise. What we want to express is that the value of the contraction factor should be adjusted according to the population size to achieve better model training.

We have removed and rephrased the statement in the manuscript regarding the strong correlation between shrinkage factor and city size to eliminate ambiguity.**]**

19. ‌**Response to [*Discussion, lines 345-351:*** *Like the contraction factor, the concept of temporal interpolation points should be introduced before this discussion.***]**‌

**Answer [**Time interpolation points refer to the time points used to calculate residuals in the Physical Information Neural Network (PINN) framework. The residual of a physical equation refers to the difference or error between the output of a neural network and the expected output of the physical equation under given input conditions. In PINN, this residual is used to evaluate whether the model's predictions conform to physical laws, i.e. the ordinary differential equations in the manuscript.

Selecting residual points (i.e. input points for calculating residuals, i.e. time interpolation points in the manuscript) is a crucial step that directly affects the learning effectiveness and final performance of the model. Therefore, when selecting time interpolation points in experiments, we must strike a reasonable balance between data length and model complexity. By optimizing the number and distribution of interpolation points, we aim to improve the accuracy and stability of the model in predicting infectious disease data.**]**

20. ‌**Response to [*Discussion:*** *Please compare a more traditional epidemiological model, such as an SIR-type model fit using least squares, and the model proposed in this paper, especially on the topics of accuracy, robustness and computational complexity. Furthermore, discuss the concrete results obtained via VOCs-INN in the context of the SARS-CoV-2 epidemic in Canada. To this end, the lines 239-253 could be moved to the discussion. In addition to being important information for epidemiologists and public health professionals, addressing these points would help to link up the discussion with the introduction.***]**‌

**Answer [**We totally agree with your suggestion. In response to your suggestions in the discussion section, we have made the following modifications and additions:

Firstly, we have added a comparison of using the least squares method to fit traditional SIR epidemiological models in the discussion section. Through comparison, we found that both the basic least squares method and the segmented least squares method did not perform as well as our proposed VOCs-INN model in terms of mean square error (MSE) and coefficient of determination (R²) evaluation metrics. This result further validates the advantages of the VOCs-INN model in terms of accuracy and robustness.

In addition, based on your suggestion, we have moved the content from lines 239-253 of the original text (which may be a description of experimental methods or preliminary results) to the discussion section to better connect with the introduction and enhance the coherence and depth of the discussion. **]**

21. ‌**Response to [*All figures:*** *Since there are multiple visually similar figures, their figure legends should be expanded where appropriate to include more information on the specific model and analysis shown.***]**‌

**Answer [**Thank you again. I have carefully reviewed the visually similar figures and expanded their figure legends to include more information on the specific models and analyses shown.**]**

22. ‌**Response to [*Fig. 1 and elsewhere:*** *Is it expected that γ(t) is nearly completely stable over the entire study duration?***]**‌

**Answer [**When fitting and analyzing the data from Italy, we set the parameter γ to a fixed value of 0.5 based on the following considerations: firstly, according to the report of the World Health Organization https://www.who.int/zh/news-room/fact-sheets/detail/coronavirus-disease-(covid-19)*,* the duration of symptoms is usually 1 to 14 days; Secondly, United Nations https://www.un.org/zh/coronavirus/covid-19-faqs documents state that individuals who have had contact with confirmed patients must undergo 14 days of self isolation. Given that the unit of removal rate γ in this article is per week, we reasonably infer and set γ to 0.5 to ensure that the model matches the actual situation. In the subsequent fitting of the data for BC province, we further defined the prior range of γ, set at 0.49 to 0.51. The analysis results show that the value of γ remains almost stable at around 0.5. Based on this discovery, in the subsequent validation of the five regions within BC province, we decided to directly set γ to 0.5 to maintain the consistency and accuracy of the model.**]**

23. ‌**Response to [*Fig. 5:*** *This figure should either be further clarified in the figure legend or simplified. It’s not clear what the different point sizes and colors mean.***]**‌

**Answer [**The different colors aim to distinguish various policies, while the varying sizes of points signify their importance—larger circles denote extremely significant policy times, whereas smaller ones indicate policies of lesser importance. Additionally, we have included nodes marking these crucial policy times in the subsequent fitting figure for enhanced clarity. We found the important time points are related to the trend of the data.**]**

24. ‌**Response to [*Fig. 6:*** *This figure could use an expanded legend.***]**‌

**Answer [**The legend in Figure 6 has been expanded accordingly.**]**

25. ‌**Response to [*Fig. 7:*** *Panels a and b are not necessary in my opinion, as all the information is contained in panel c.***]**‌

**Answer [**Panels a and b in Figure 7 have been removed as suggested.**]**

26. ‌**Response to [*Fig. 7-12:*** *Consider adding some important interventions presented in Fig. 5 as vertical lines to better understand some of the fluctuations.***]**‌

· **Answer [**I have optimized Fig. 5 based on your feedback. In order to more intuitively demonstrate the impact of these time points on the results, I have added vertical lines in the subsequent fitting graph. In this way, readers can have a clearer understanding of the correlation between data fluctuations and these intervention measures.**]**

27. ‌**Response to [*Fig. 9:*** *The legend refers to shaded areas, which are however not visible by eye without zooming in excessively. If I understand correctly, this is due to the shaded areas being extremely small. It would be good to point out that these areas are very small in the legend to avoid confusion.***]**‌

**Answer [**Thank you for your feedback on the visibility of the shaded areas in Fig. 9. To address this issue, I have included enlarged versions of these shaded areas in the supplementary materials as Fig. S9 and Fig. S10. **]**

28. ‌**Response to [*Fig. S2, S4-S9:*** *Please provide a key for the abbreviations used as figure titles (IH, FH, NH, VCH, VIH). Their meanings can be guessed using Fig. S1, but it should be part of the figure legends.***]**‌

**Answer [**We appreciate your suggestion.You are correct that the meanings of the abbreviations (IH, FH, NH, VCH, VIH) used as figure titles can be inferred from Fig. S1 in the supplementary materials. In order to improve clarity, I have listed the full names and corresponding abbreviations of the five health bureaus in the comments Table 2, instead of adding them to the legend of Figure S1 as originally planned. This adjustment ensures that readers can easily understand the specific meanings of these abbreviations without referring to additional materials.**]**

**In addition to the specific responses above, I have also made detailed revisions and improvements in multiple areas based on the overall structure and content of the paper to enhance its quality and readability. The specific details of these modifications have been annotated in the corresponding positions in the manuscript.**
