# Supplementary material for: Using a multi-strain infectious disease model with physical information neural networks to study the time dependence of SARS-CoV-2 variants of concern
Source: PLoS Comput Biol. 2025 Feb 14;21(2):e1012778. doi: 10.1371/journal.pcbi.1012778 (PMC11828408; doi:10.1371/journal.pcbi.1012778)
Supplement: Attachment — Submitted filename: review_comments_r1.docx [file pcbi.1012778.s016.docx]

**Review comments (Revision 1)**

Li et al., *Using a multi-strain infectious disease model with physical information neural networks to study the time dependence of SARS-CoV-2 variants of concern*

Overall, I find the manuscript significantly improved, and I appreciate the effort that went into incorporating the suggested changes. There are still some aspects that I think make it hard to understand and evaluate the work.

Although I find the whole paper a challenging read, I feel it reads reasonably well throughout the introduction, methods and results sections.

My main concern is the discussion: While it is now more extensive, the first two sections, titled “Comparison with nonlinear least squares method” and “The impact of physical information” read like an extension of the results section and provide little context or discussion on how to evaluate these results. I appreciate the added information, but feel that the results and technical aspects contained in these sections should be moved to the results and methods sections, as appropriate, and they should be discussed and contextualized in the discussion without the precise technical detail. As it stands, I find it very hard to gauge what the main message of the discussion is, and this hampers the main message of the paper as a whole.

Here are some other, minor comments that I feel should be addressed:

- Please clarify the scale of the y-axis in Figures 3 and 5 (left y-axis for Fig 5). Which units are shown here?
- Line 227-228: "Also, THEY confirm the reasonableness and creditability of our parameter estimations."
- Check the title of the figure legend of Figure 8 for grammatical correctness: "Forecasted the transmission rates and the removal rates utilizing the VOCs-INN approach." I would remove both occurrences of "the".
- Figure 8 legend: I find this legend quite confusing to read. What is an “actual prediction scenario” in this context? I wonder whether the whole Figure 8 is necessary to include, as it only plays a role as a stepping stone for Figures 9 and 10. I would consider dropping this figure entirely (or moving this to the supplementary material), but leave this decision up to the authors. Personally, I think Figure 8 is currently quite confusing.
- Line 360: Check for grammatical correctness. Do you mean “ReproducING the competition and coexistence dynamics”?
- Line 392: “The” in “Firstly, The application …” should be lowercase.
- Lines 392-411: I appreciate the added explanation of the contraction factor and time interpolation points, but I disagree with the way they are integrated in the discussion. In particular, I would have expected an assessment if and how these factors impact the robustness and precision of the results obtained, which is currently absent.
- Table 4: How can R^2^ have a value of -7.2195 (Middle column)?
