# Supplementary material for: Using a multi-strain infectious disease model with physical information neural networks to study the time dependence of SARS-CoV-2 variants of concern
Source: PLoS Comput Biol. 2025 Feb 14;21(2):e1012778. doi: 10.1371/journal.pcbi.1012778 (PMC11828408; doi:10.1371/journal.pcbi.1012778)
Supplement: Attachment — Submitted filename: Response_to_Reviewer_2_auresp_2.docx [file pcbi.1012778.s017.docx]

**PCOMPBIOL-D-24-01438R1, Using a multi-strain infectious disease model with physical information neural networks to study the time dependence of SARS-CoV-2 variants of concern,**

**by Li, Chen and Liu et al**

**Authors’ responses to the comments and suggestions of Reviewer #2**

We first of all would like to thank you again for careful reading of our revised manuscript and for all the constructive critiques and helpful suggestions, which have been instrumental improved the quality of our manuscript. In the latest modified version, we have carefully considered each of your points. Below are our detailed responses and the modifications we have made.

Your comments are in italic and our responses are in normal text, for the ease of reading.

***main concern***

1. ‌**Response to [***My main concern is the discussion: While it is now more extensive, the first two sections, titled “Comparison with nonlinear least squares method” and “The impact of physical information” read like an extension of the results section and provide little context or discussion on how to evaluate these results. I appreciate the added information, but feel that the results and technical aspects contained in these sections should be moved to the results and methods sections, as appropriate, and they should be discussed and contextualized in the discussion without the precise technical detail. As it stands, I find it very hard to gauge what the main message of the discussion is, and this hampers the main message of the paper as a whole.***]**‌

**Answer:** We totally agree with your comment. We acknowledge that these added subsections read more like an extension of the results section and did not provide the anticipated depth of analysis and contextualization. In response to your suggestion, we have made the following revisions:

**· Restructuring Content**: We have moved the technical details and specific results from these two subsections to the appropriate position within the Results and Methods sections.

**· Enhancing the Discussion Section**: In the Discussion section, we have thoroughly revised and rewritten the content to provide a comprehensive analysis and contextualization of the results. By removing the precise technical details from this section, we have been able to create a clearer and more coherent narrative that highlights the main messages of our study.

**· Combining Discussion and Conclusion Sections**: To streamline the manuscript and enhance readability, we have combined the Discussion and Conclusion sections into a single chapter. This unified section now serves to summarize the key findings of our research, reiterate its importance, and provide a concise conclusion that ties all the elements of the paper together. Once again, thank you for your invaluable feedback.

***minor comments***

2. ‌**Response to [***Please clarify the scale of the y-axis in Figures 3 and 5 (left y-axis for Fig 5). Which units are shown here?***]**‌

**Answer:** We appreciate your suggestion. The units displayed on the y-axis for both Figure 3 and the Figure 5 are individuals. We apologize for any confusion this may have caused. To ensure transparency and clarity, we have now explicitly stated the units in the captions of both figures. We believe that these additions will aid readers in understanding the scale and units used in these figures.

3. ‌**Response to [***Line 227-228: "Also, THEY confirm the reasonableness and creditability of our parameter estimations.*"**]**‌

**Answer:** Thank you for pointing out the oversight. We have corrected the sentence by adding the missing word "they" to ensure clarity and grammatical accuracy.

4. ‌**Response to [***Check the title of the figure legend of Figure 8 for grammatical correctness: "Forecasted the transmission rates and the removal rates utilizing the VOCs-INN approach." I would remove both occurrences of "the".***]**‌

**Answer:** Thank you for your suggestion. We have revised the title of the figure legend for Figure 8 by removing both occurrences of "the" to improve grammatical correctness.

5. ‌**Response to [***Figure 8 legend: I find this legend quite confusing to read. What is an “actual prediction scenario” in this context? I wonder whether the whole Figure 8 is necessary to include, as it only plays a role as a stepping stone for Figures 9 and 10. I would consider dropping this figure entirely (or moving this to the supplementary material), but leave this decision up to the authors. Personally, I think Figure 8 is currently quite confusing.***]**‌

**Answer:** Thank you very much for your valuable suggestions. We fully understand your concerns regarding the clarity of the legend in Figure 8 and the potential confusion caused by the term "actual prediction scenario." To address this, we have revised the legend of Figure 8 by removing the word "actual," simplifying it to "prediction scenario" to more intuitively convey the purpose and context of the figure. Additionally, to streamline the main manuscript and address the confusion you mentioned, we have moved Figure 8 to the supplementary material.

6. ‌**Response to [***Line 360: Check for grammatical correctness. Do you mean “ReproducING the competition and coexistence dynamics”?***]**

**Answer:** Thank you for your attention to detail. We have corrected the grammatical error by changing "Reproduce" to "Reproducing" in line 360.

7. **Response to [***Line 392: “The” in “Firstly, The application …” should be lowercase.***]**

**Answer:** Thank you again. We have adjusted the capitalization by changing "The" to lowercase in line 392.

8. **Response to [***Lines 392-411: I appreciate the added explanation of the contraction factor and time interpolation points, but I disagree with the way they are integrated in the discussion. In particular, I would have expected an assessment if and how these factors impact the robustness and precision of the results obtained, which is currently absent.***]**

**Answer:** Thank you for your constructive feedback. We have taken your suggestions into consideration and have revised our manuscript accordingly. Specifically, we have moved the additional explanation of the contraction factor and time interpolation points to the Results section. Furthermore, in the Discussion section, we have now included an assessment of how these factors impact the robustness and accuracy of the obtained results. To support this assessment, we have conducted additional analyses with varying contraction factors and time interpolation points, observing the fitting performance of VOCs-INN and the ODE inverse solutions. The corresponding figures have been included in the supplementary material.

9. **Response to [***Table 4: How can R^2^ have a value of -7.2195 (Middle column)?***]**

**Answer:** Thank you for your inquiry regarding the *R*² value in Table 4. While it is true that R² values typically range between 0 and 1, this assumes that the model provides a reasonable fitting to the data. In this particular case, the *R*² value of -7.2195 indicates an extremely poor fitting of the model to the data. This negative value arises due to the significant discrepancy between the observed data and predicted values, indicating that the parameter estimates for the model are poor, leading to inaccurate predictions.

**In addition to the specific responses above, we also made detailed revisions and improvements. The specific details of these modifications have been annotated in the corresponding positions in the latest revised vision.**
